# Pullulan Coating Preserves High Conductivity in Cable Bacteria Wires

**DOI:** 10.1021/acsabm.5c02310

**Published:** 2026-02-11

**Authors:** Anastasia Gerzhik, Dmitrii Pankratov, Silvia Hidalgo Martinez, Filip J. R. Meysman, Andreas Offenhäusser, Dirk Mayer

**Affiliations:** 1 Institute of Biological Information Processing (IBI-3), Forschungszentrum Jülich, Jülich 52428, Germany; 2 Faculty I, RWTH Aachen University, Aachen 52062, Germany; 3 Geobiology Research Group, Department of Biology, 26660 University of Antwerp, Universiteitsplein 1, Wilrijk 2610, Belgium

**Keywords:** biobased electronics, cable bacteria, conductance, pullulan, protective coating

## Abstract

The greening of electronics remains a grand societal challenge, with no radical improvement within sight. Sustainable solutions for electronics, such as biobased and transient materials, are hence receiving growing attention. Presently, there are no biobased alternatives to conventional conductors such as metals and organic polymers, as their conductivity is too low. The discovery of cable bacteria, which are filamentous microorganisms capable of conducting electricity over centimeter-scale distances, has the potential to change this. In cable bacteria, conductivity occurs through thin wires embedded in the cell envelope, displaying conductivities comparable to those of the best highly doped organic polymers. However, exposure to ambient air leads to a gradual loss of their conductivity. To enhance stability, a bioderived protective coating could be useful, thus retaining a fully biobased system. To this end, we investigated pullulan, a polysaccharide polymer primarily used in food packaging that is known for its excellent oxygen-barrier properties. Cable bacterium filaments protected with a film derived from a 10 wt % pullulan solution exhibited a 10-fold increase in conduction stability under ambient conditions compared to uncoated controls. Reducing ambient moisture also preserved the long-term conductivity of the cable bacteria, even in the absence of a protective coating, indicating that humidity plays a critical role in conductance deterioration. Our findings provide an important step toward further technological implementation of the highly conductive wires of cable bacteria and offer practical guidelines for developing biobased coatings for O_2_-sensitive materials in electronics, thus contributing to the advancement of next-generation green technologies.

## Graphical abstract

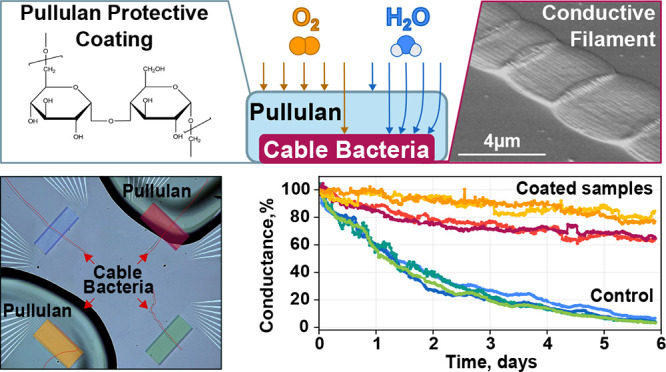

## 1 Introduction

Cable bacteria are long, filamentous microbes, which are capable of sending internal electrical currents over centimeter-long distances.^1–3^ These currents are guided through a network of parallel fibers (for *Ca.* Electrothrix gigas there are ∼60 fibers with ∼50 nm diameter, >10 mm long) that are embedded in the cell envelope (Figure 1).^4,5^ These fibers act as a powerline network,^6,7^ and display a very high conductivities, reaching up to 564 S/cm,^6,8,9^ which is comparable to doped silicon^10,11^ or highly doped organic polymers.^12–14^ Such extreme conductivity is a remarkable property for a biological material, and so, these bacterial fiber structures have emerged as a compelling candidate material for the development and investigation of nature-inspired, sustainable bioelectronic systems.^8^

**Figure 1:**
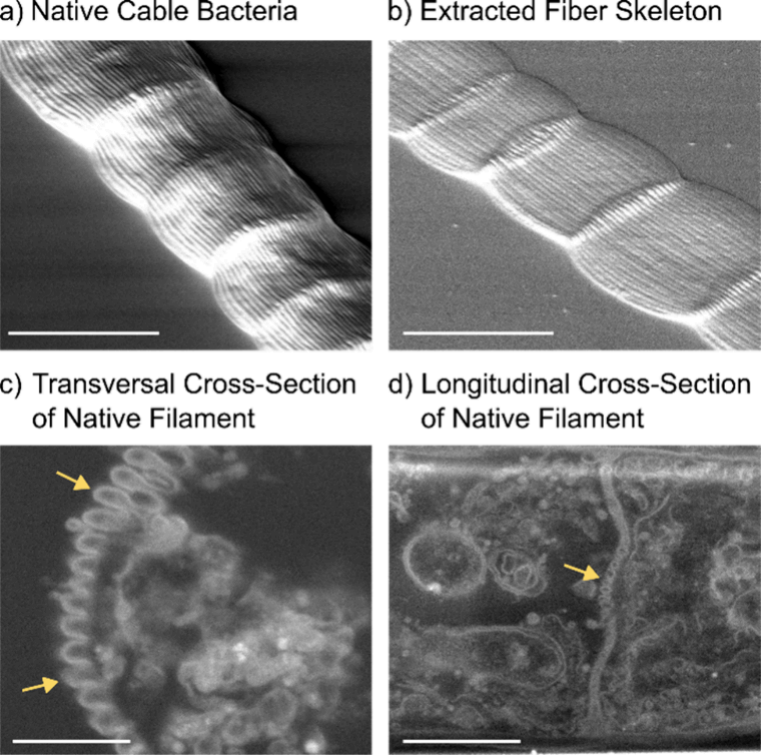
Scanning electron microscopy images showing the morphology of cable bacteria. All filaments belong to the group *Ca*. Electrothrix gigas. (a) Native filament. Scale bar: 4 μm. (b) Fiber skeleton obtained after removal of membranes and cytoplasm (extraction with SDS and EDTA). Scale bar: 4 μm. (c) Transversal FIB cross section of a native filament. The arrows indicate the position of the ridge compartments with the black core representing the conductive fiber. Scale bar: 500 nm. (d) Longitudinal FIB cross section of a native filament. The arrow indicates the cell–cell junction. Scale bar: 1 μm.

Currently, the charge transport mechanism in the fibers remains enigmatic, but it is clearly distinct from the conventional multisite hopping as seen in other conductive protein structures, due to it its very low reorganization energy and the absence of any redox signature.^7,8,15,16,54^ Recent Raman microscopy analysis suggests that the fibers embed a bioinorganic compound that resembles a nickel bis­(1,2-dithiolene) organic framework,^17–19^ but the exact molecular structure remains presently unresolved. Still, once the molecular structure and biosynthesis procedure are better understood, the cable bacteria have the potential to provide a novel, biobased, highly conductive organic material that holds particular promise for biobased flexible and transient electronics. For instance, biobased conductive inks can be designed to decompose into untraceable residues upon demand (after a stable period of device operation). Accordingly, this would provide a substantial environmental advantage over metal-based inks, particularly silver-based formulations, whose use is associated with ecotoxic risks when released into the environment.^20^ Biobased electronic materials attract a growing interest due to the urgent need to reduce the generation of electronic waste, particularly in view of the projected near-term rise of short-lived and disposable electronic devices.^21^

However, one bottleneck for widespread application and future deployment of the cable bacteria-inspired material is the sensitivity of the fiber conductivity toward ambient air. In the presence of oxygen, the conducting fibers undergo a gradual loss of conductivity over a period of several hours.^5,6^ In contrast, when examined in a vacuum chamber or under an inert N_2_ atmosphere, the fibers preserve their conductive properties for months.^6,9^ This conductance degradation phenomenon is not yet fully understood but has been linked to reactive oxygen species that are generated upon exposure to O_2_.^6,22^ This response is thought to be related to the specific metabolism of cable bacteria, which live in the anoxic part of aquatic sediments.^22,23^ Therefore, cable bacteria are not adapted to prolonged O_2_ exposure, which causes oxidative stress, and this could lead to the degradation of their conductive structures. The degradation of conductivity can be significantly slowed by an extraction of cell membranes and cytoplasm by sequential SDS and EDTA treatments.^6^ The remnants of the cable bacteria left after the extraction are termed fiber skeletons, as they retain the conductive fiber structures (Figure 1b).^4,6^ Interestingly, these fiber skeletons remain equally conductive as the native (unextracted) filaments. Yet, even for fiber skeletons, oxidative aging leads to a loss of conductivity over a time scale of 1–7 days, suggesting irreversible damage to the conductive fiber structures.

The sensitivity of conductance toward oxygen can limit the practical application of bioelectronic materials inspired by cable bacteria. Here, we propose to remediate this issue by implementing a protective coating that meets the primary requirements of low oxygen permeability, alongside ease of application, to allow for scalable fabrication in the future.

Conformal or thin polymeric film coatings have been used commercially for over 60 years to protect sensitive electronic components from aggressive environments.^24^ Over this period, a variety of coating methods have been developed, spanning from conventional spray coating and epoxy resin molding to technologically demanding thermal vacuum evaporation and physical vacuum deposition.^24–26^ Coatings should reliably adhere to the substrate and effectively provide a protective functionality determined by the conditions under which the device is used. Among these, oxygen-barrier formation and moisture resistance are two important demands for modern electronic components.^24,26^

At present, integrated circuits are typically encapsulated in plastic cases and embedded in epoxy resins, which have proved to be cost-effective and reliable for most of the standard use cases.^27,28^ For applications that require enhanced gas barrier performance, such as OLED passivation, the leading approach is the fabrication of multilayered barrier films comprising alternating organic and inorganic layers.^25,26^ Yet, these methodologies do not fulfill the criteria for sustainability, as epoxy resins are synthesized from fossil-based compounds,^29,30^ while coating processes utilized in OLED manufacturing generate significant greenhouse gas emissions and nanowastes.^31^

Research on sustainable protective biopolymer coatings is evolving rapidly, driven by the need to reduce the environmental impact of existing fabrication processes.^21,30–33^ In this context, polysaccharides such as cellulose, starch, and chitosan are employed for barrier packaging films, owing to their biodegradability, renewability, and widespread availability.^34^ Among these biopolymers, pullulan is notable for its exceptional oxygen-barrier properties—comparable to those of some synthetic polymers—in addition to its excellent film-forming capabilities, edibility, and nontoxic nature.^35–38^ Pullulan is a water-soluble polysaccharide produced by a yeast-like fungus, which comprises a linear polymer composed of maltotriose units linked via α-(1, 6) glycosidic bonds.^36^ Pullulan forms transparent films that effectively impede the transmission of oxygen.^39^ For instance, a film derived from a 5 wt % aqueous pullulan solution exhibits an oxygen permeability of only 6.3 L·μm·m^–2^·day^–1^·atm^–1^.^37^ For comparison, polyethylene (PE), the most widely used plastic, displays a more than 10-fold higher oxygen permeability, ranging between 50 and 200 L·μm·m^–2^·day^–1^·atm^–1^.^40^ Up to now, pullulan has been successfully used for the preservation of oxygen-sensitive bacteriophages^41^ and vaccines,^42^ and its properties can be tuned to improve barrier performance and modify mechanical characteristics.^43–48^

The application of pullulan has been explored before in the field of transient electronics, e.g., it has been employed as a biodegradable membrane in vibration sensors,^49^ as a water-processable binder and separator in supercapacitors,^50^ as a flexible triboelectric layer in nanogenerators,^51^ and as a sacrificial, water-soluble support transferring electronic decals to skin.^52^ Yet, in these studies, pullulan always serves as a substrate or matrix, and so, its usage is driven by its water solubility and biodegradability. Here, we exploit its excellent oxygen-barrier properties, thus targeting a functional coating that could enhance the stability and longevity of electronic components. We experimentally investigate whether the conductance of fiber skeletons derived from cable bacteria is preserved by a pullulan-based coating, with the long-term vision to establish a fully biobased and sustainable next-generation electronic system.

## 2 Materials and Methods

### 2.1 Cable Bacteria Culturing and Fiber Skeleton Extraction

Cable bacterium filaments were derived from clonal cultures of *Candidatus* Electrothrix gigas strain JX3-16, obtained according to a previously reported protocol.^53^ Clonal cultures are grown within autoclaved sediment and contained a single strain of cable bacteria. For the extraction procedure, a sediment core containing a clonal enrichment was transferred into an anaerobic chamber (Coy Laboratory Products, Inc., Grass Lake, Michigan, USA) filled with a 95/5% mixture of N_2_ and H_2_ gases, with [O_2_] < 20 ppm, RH = 55–60%, and *T* = 22–25 °C. Individual cable bacterium filaments were retrieved from the sediment with a custom-made glass hook fabricated from Pasteur pipettes.^7^ After a sequence of washes in purified water (ISO 3696 grade 1, Milli-Q), the filaments underwent a chemical extraction procedure with a 1% (w/w) aqueous solution of sodium dodecyl sulfate (SDS), followed by a wash with 1 mM EDTA, pH 8 for 10 min at RT each.^4^ This extraction produces a so-called “fiber skeleton”, which still contains the conductive fibers embedded onto a supporting carbohydrate sheath but removes most of the other cell material (membranes and cytoplasm), see Figure 1b.^7^ Importantly, the extraction procedure does not affect the fiber conductivity,^6^ and so, the fiber skeletons provide a suitable material to probe the impact of external factors like temperature and ionic strength on the fiber conductivity.^9,54^ After the extraction, fiber skeletons were washed six times in Milli-Q water and individually placed on top of microfabricated chips containing gold electrodes for electrical characterization (Figure 2). The aforementioned steps were all performed in an anaerobic chamber. Afterward, the samples were transferred for pullulan coating to a different glovebox with an inert Ar atmosphere (200B, M. Braun Inertgas-Systeme GmbH, Garching, Germany). A vacuumized container (Zwilling J. A. Henckels AG, Solingen, Germany) was used for the transfer so that the samples were not exposed to ambient air prior to the stability measurements.

**Figure 2:**
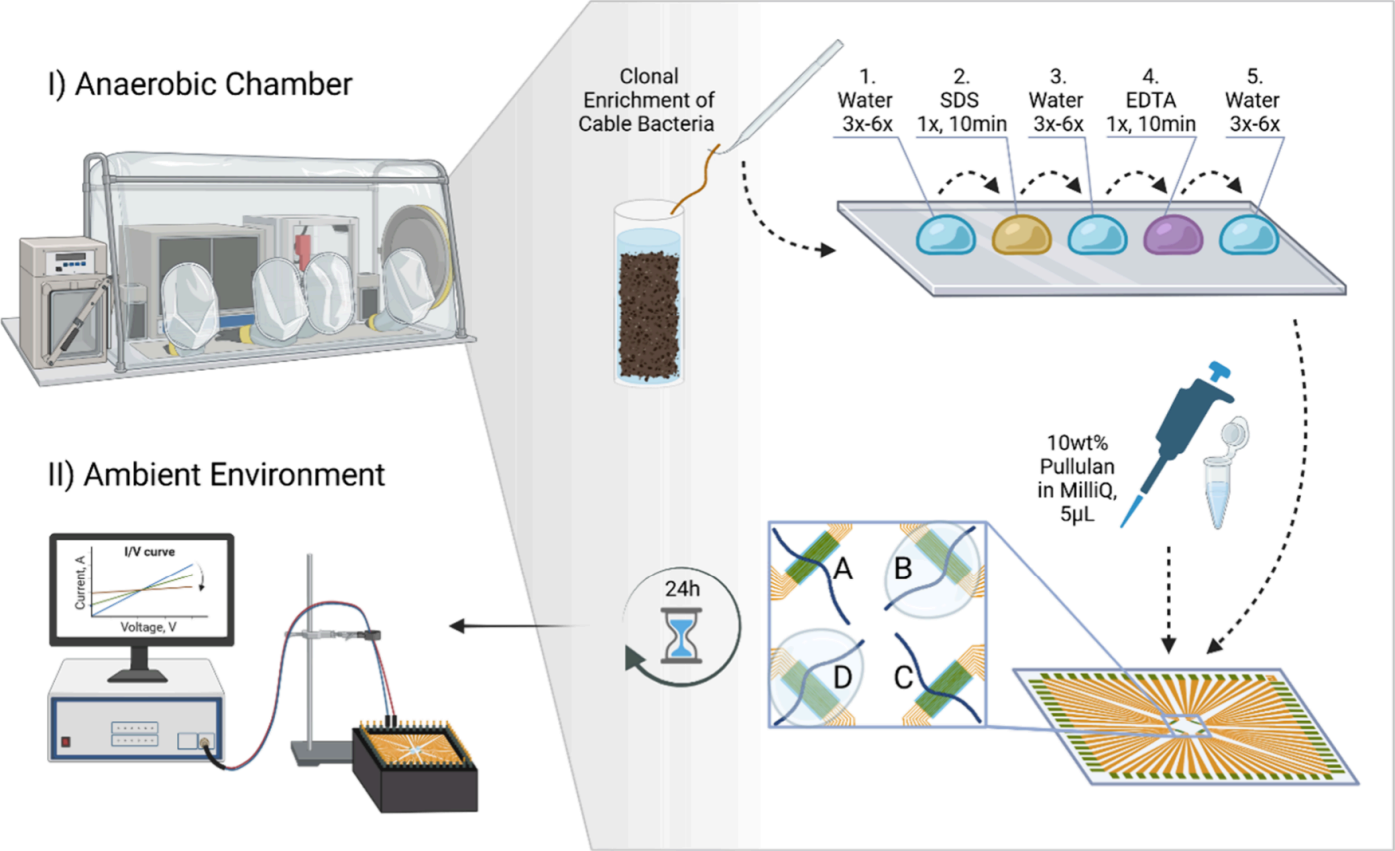
(I, II) Experiment workflow showing the preparation steps performed under anaerobic conditions. Cable bacterium filaments are extracted, placed on gold MEA, covered by pullulan solution, dried, and characterized by dynamic *I*/*V* profiling in an ambient atmosphere. The MEA design comprises four distinct electrode pads, labeled A–D, each having 17 individually addressable electrodes. Half of the electrode pads (B and D with fiber skeleton segments T1–T4) were coated with 5 μL of a 10 wt % aqueous solution of pullulan, while the other half (A and C with segments C1–C4) remained uncoated for control. Created with permission from BioRender.com/y8ov550.

### 2.2 Fabrication of Gold Microelectrode Arrays

Microelectrode arrays (MEAs) were fabricated on n-doped silicon wafers with a resistivity of 5–10 Ohm·cm (Siegert wafer GmbH, Aachen, Germany). Thermal oxidation of wafers was performed using the wet oxidation process at 1050 °C for 190 min (CLV200, Centrotherm Systemtechnik GmbH, Brilon, Germany) to attain a 1 μm silicon oxide layer. The metal feedlines were patterned by forming the resist mask, which involved stacking of the lift-off resist LOR 3b (MicroChem Corp, Massachusetts, USA) and the light-sensitive resist AZ nLOF 2020 (MicroChemicals, Ulm, Germany). The latter was patterned using the Mask Aligner MJB4 (SUSS MicroTec SE, Germany) with a mounted i-line and 33% grayscale filters at an estimated irradiance of 6 mW/cm^2^. After resist development, a metal stack of 20 nm titanium (Ti), 120 nm gold (Au), and 10 nm titanium (Ti) was deposited by electron-beam evaporation (Pfeiffer PLS 570, Pfeiffer Vacuum, Asslar, Germany). Gold was selected as the primary conducting material due to its high electrical conductivity and biocompatibility,^55^ while titanium serves as an adhesion layer to ensure the stability of the gold film on the substrate.^56^ In the next step, the resist was lifted off together with the excess metal by soaking the wafer in acetone (Technic Inc., Rhode Island, USA). To restrict the exposed metal area to the size of the actual microelectrodes and to protect the metal leads, the MEAs were passivated with an 800 nm stack of alternating SiO_2_ (200 nm) and Si_3_N_4_ (100 nm) layers (ONONO) via plasma-enhanced chemical vapor deposition (PECVD) (SENTECH Instruments GmbH, Berlin, Germany). On top, a finishing 40 nm Ta_2_O_5_ film was grown via atomic layer deposition (ALD) by a FlexALII machine (Oxford Instruments, Great Britain) to close pinholes in the passivation film. To enable access to the gold electrodes, the passivation and the top Ti layer were selectively etched using reactive ion etching (RIE) at 150 W RF power and a gas mixture of CHF_3_/CF_4_/Ar/O_2_. After this step, the RIE-protection resist (AZ nLOF 2020) was stripped, and another protection resist was deposited for the final dicing of the wafer into individual 24 × 24 mm chips. The design of the MEAs was developed in the layout editor CleWin (WieWeb software, Hengelo, The Netherlands), comprising four electrode sets designated A, B, C, and D. Each set, indexed A–D, contains 17 individually addressable electrodes 8 μm in width and 900 μm long. The electrodes A–D are spaced by 6, 8, 10, and 12 μm, respectively. The exact layout is shown in Figure S10 and Table S2 in the Supporting Information.

Before the experiments, MEAs were cleaned in acetone and 2-propanol (Technic Inc., Rhode Island, USA). Subsequently, they were exposed to an oxygen plasma in a GIGA batch 310 M system (PVA TePla AG, Wettenberg, Germany) and finally treated with piranha solution (i.e., a mixture of 30% H_2_O_2_ and 96% H_2_SO_4_ (Technic Inc., Rhode Island, USA) solutions in proportion 2:1) for 10 min to fully remove all resist residuals.

### 2.3 Pullulan Coating

Pullulan is a highly hydrophilic polysaccharide and, hence, can be easily dissolved in water. Here, pullulan (Merck KGaA, Darmstadt, Germany) was prepared in a 10 wt % solution with Milli-Q water by vortexing for 5 min, followed by O_2_ degassing in the Ar atmosphere of the glovebox for at least 1 h. The 10 wt % concentration was chosen based on known examples from literature, where the formed pullulan film efficiently preserved properties of vaccines or bacteriophages.^41,42^

In the protective treatment, 5 μL of pullulan solution was casted onto the two fiber skeletons that had previously been placed on the electrodes, while two others were left as uncoated control filaments (Figure 2). Afterward, the samples were left to dry inside the glovebox for 24 h to form a solid protective film. The pullulan film thickness was evaluated at the end of the experiment (Dektak 150 Surface Profiler, Veeco Instruments Inc., Tucson, AZ, USA) and was 13 μm at its thinnest point in the center and increased up to 67 μm on the edges (Figure S2).

### 2.4 Electrical Characterization

Subsequent to the pullulan film drying in the glovebox, the MEAs with deposited fiber skeletons were transferred to the ambient air (*T* = 19–23 °C, RH = 35–50%). The conductance was assessed by consecutive *I*/*V* profiling with the resistance calculated from the slope of the *I*/*V* curve. For each of the four fiber skeletons examined, two distinct segments were characterized, resulting in a total of eight segments. The pullulan-coated segments are named T1–T4 (treatments), while the control segments are labeled C1–C4. To ensure that the pullulan does not contribute to conductance, the control blank MEA with pullulan film was characterized (Figure S7).

Continuous *I*/*V* curve recording was performed using a PalmSens4 potentiostat with an MUX8 multiplexer (Palmsens BV, Houten, The Netherlands) in a two-probe configuration applying a voltage bias from −200 to +200 mV (2 mV steps at 5 mV/s scan rate). To establish a connection with the MEA, the chip was mounted in a holder with spring contacts placed over the electrodes. The contacts lead to the pins of the electric socket at the other end, enabling wiring to the terminals of the potentiostat multiplexer. The eight sample segments were addressed sequentially, with a 5 s interval between successive measurements on distinct segments and 22 min interval between two measurements on the same segment. The overall experiment duration was 8200 min (5.7 days). The *I*/*V* curves of fibers skeleton segments were highly linear over the voltage bias range implemented (Figure 3b) and can be fitted using the equation: *I* = *k*_fit_·*V + c*_fit_, with *I* is the current, *V* is the bias voltage, *k*_fit_ is the slope corresponding to the conductance, reciprocal to the resistance: *k*_fit_ = *G* = 1/*R*, and *c*_fit_ is the offset coefficient (caused by the measurement system bias, but generally close to 0).

To express how the conductance *G* of a given segment varied with time *t* during the experiment and simplify comparison between different segments, the residual conductance is introduced:

$$\mathrm{residual}\;\mathrm{conductance}=\frac{G(t)}{G_{0}}\cdot 100\%=\frac{R_{0}}{R(t)}\cdot 100\%$$

In this, *G*_0_ and *R*_0_ are the initial values of the conductance/resistance of the sample at the beginning of the experiment, respectively. In addition, to be able to quantitatively compare the degradation behavior of different samples, the conductance loss rate κ was calculated as the percentage of conductance the sample loses per unit of time:

$$\kappa =\frac{G\left(t_{i}\right)-G\left(t_{i+1}\right)}{G\left(t_{i+1}\right)\cdot \left(t_{i+1}-t_{i}\right)}\cdot 100\%=\frac{R\left(t_{i+1}\right)-R\left(t_{i}\right)}{R\left(t_{i}\right)\cdot \left(t_{i+1}-t_{i}\right)}\cdot 100\%$$

To avoid noise amplification, the *R*(*t*) data were first smoothed using the lowess (locally weighted scatterplot smoothing) function from the statsmodels Python package (with 0.07 as smoothing parameter value). The lowess algorithm is a least-squares regression method that fits the scattering data without using the predetermined equation^57^ and is more stable to outliers compared to other smoothing functions like the binomial filter or moving average.

To distinguish the intrinsic filament resistance *R*_*i*_ from the contact resistance of the sample to the electrodes *R*_c_, additional four-probe measurements were executed on every second day of the experiment (Keithley 4200 parameter analyzer, Tektronix, Munich, Germany). In this approach, the current is forced over the two outer electrodes, while the voltage is measured from the inner electrodes—the same electrode pair that is used for the two-probe stability measurements. The slope of the four-probe *I*/*V* curve represents the intrinsic resistance *R*_4p_ *= R*_*i*_,^58,59^ and so, the contact resistance *R*_c_ can be estimated from the difference between two-probe and four-probe resistances. This approach also enables the estimation of the intrinsic fiber conductivity using the following expression:^9^

$$\sigma_{F}=\frac{L}{R_{i}\cdot N_{F}\cdot A_{F}}=\frac{L}{R_{i}\cdot N_{F}\cdot \pi \cdot (r_{F}{)}^{2}}$$

Here, *L* represents the length of the fiber skeleton segment between the internal (potential) electrodes used for the four-probe recording, *R*_*i*_ is the intrinsic sample resistance estimated from the linear fit of the four-probe *I*/*V* curve, *A*_F_ is the cross-sectional area of one fiber, *N*_F_ and *r*_F_ represent the number and radius of the conductive fibers, which are estimated as 60 fibers and 25 nm, respectively, for the examined *Ca.* Electrothrix gigas filaments.

### 2.5 Statistical Analysis

The derived conductance loss rates κ (*N* = 372 for each segment) were used in the statistical pairwise comparison of the pullulan-coated and control samples. The normality of the distribution of the conductance loss rates was examined using the Shapiro-Wilk test. This showed that the resulting data distributions could not be considered normal (*p*-value <0.05). Therefore, nonparametric Dunn’s test with Bonferroni correction was implemented for pairwise comparisons of segments. Those were performed after the application of Kruskal–Wallis’s criteria, the nonparametric equivalent of ANOVA, which indicated the presence of a statistically significant difference in at least one of the group pairs (*p*-value <0.05).

### 2.6 Scanning Electron Microscopy (SEM), Focused Ion Beam Scanning Electron Microscopy (FIB-SEM), and Light Microscopy

SEM imaging of cable bacteria filaments was performed on a Magellan 400L XHR Scanning Electron Microscope (FEI Company, Hillsboro, Oregon, USA). For this, native filaments or fiber skeletons were placed on a Si substrate with 100 nm of SiO_2_ and imaged using a secondary electron (SE) detector without prior metallization since the samples possess sufficient conductivity to spread electrons and prevent charging. The accelerating voltage was 1 kV at 50 pA beam current, 4.1 mm working distance, and 52° tilt.

The FIB cross sections were obtained by chemically fixing the native cable bacteria based on a previously reported protocol.^4^ The precise procedure for the chemical fixation and FIB-cutting techniques employed in this study are found in the Supporting Information. The resulting cross sections were imaged using a SEM FEI Helios Nanolab 600 (Hillsboro, Oregon, United States) with a backscattered electron detector at 3 kV accelerating voltage, 86 or 690 pA beam current, 4.1 mm working distance, and 52° tilt.

Light microscopic images of MEAs with fiber skeletons coated with pullulan were collected at the end of the stability experiment (day 6) using a Keyence VK-X 100 microscope (Keyence, Osaka, Japan).

### 2.7 Evaluation of the Relative Humidity Influence on the Cable Bacteria Degradation

After the end of the main experiment, the uncoated control segment C1 was utilized to further investigate the effect of the relative humidity on the conductance. To achieve this, the MEA chip was placed inside a desiccator containing 200 g of silica gel (Merck KGaA, Darmstadt, Germany). The relative humidity inside the desiccator decreased from an average of 40% ambient to below 10% (limit of detection) after 1000 min, as registered by the ThermoPro TP357 sensor (ThermoPro, Lawrenceville, Georgia, USA). Later control measurements in the same desiccator without cable bacteria enabled an estimation of the decrease in humidity below 10% after 7 h, 6% after 1 day, and 1.5% after 5 days measured with a humidity sensor Trotec BL30 (Trotec GmbH, Heinsberg, Germany). The corresponding [O_2_] values were monitored using the oxygen sensor ECO 410 (GSG Geologie-Service GmbH, Würzburg, Germany) and remained at an ambient level of 21% across all RH values. To monitor the sample’s resistance, continuous *I*/*V* profiling was performed as done before using the PalmSens4 potentiostat.

## 3 Results and Discussion

To evaluate the performance of pullulan as a bioderived coating for oxygen-sensitive electronics, we applied it to fiber skeletons of cable bacteria—thin sheaths (∼300 nm thickness) that embed the conductive fibers responsible for long-range electron transport. In the presence of oxygen, the charge transfer in these fiber skeletons is known to be progressively impaired, eventually resulting in full conductivity loss.^6^ Pullulan coating was therefore expected to improve the temporal stability of the sample conductance with regard to exposure to air containing O_2_. To test this hypothesis, our initial experimental steps were carried out in an anaerobic chamber, where cable bacteria were first extracted using the SDS/EDTA protocol, resulting in fiber skeletons that were placed on the gold microelectrode arrays (MEAs), as shown in Figure 2. Both, pullulan treated and noncoated control samples were prepared on the same MEA, as to ensure that all segments were exposed to exactly the same environmental conditions throughout the experiment (Figure 3a). To achieve the pullulan film formation, the samples were left to dry after the solution casting in the inert atmosphere for a period of 24 h. Subsequent to the drying process, the MEAs were transferred to ambient air, and their stability was assessed by dynamic *I*/*V* recording (Figure 3b). It is worth noting that the pullulan film did not always fully cover the filament (e.g., Figure 3a, filament B), leaving part of it exposed to ambient atmosphere and allowing conductance degradation in this region. However, the part of the filament that was electrically characterized (red zone in Figure 3a) remained centrally located under the pullulan cover far from the edge of the pullulan film (∼150 μm). We therefore surmise that the presence of the filament did not impair O_2_ protection (e.g., by allowing O_2_ to penetrate along or within the thin bacterial filament, which has a cross section of only 4 × 0.3 μm^2^).

**Figure 3:**
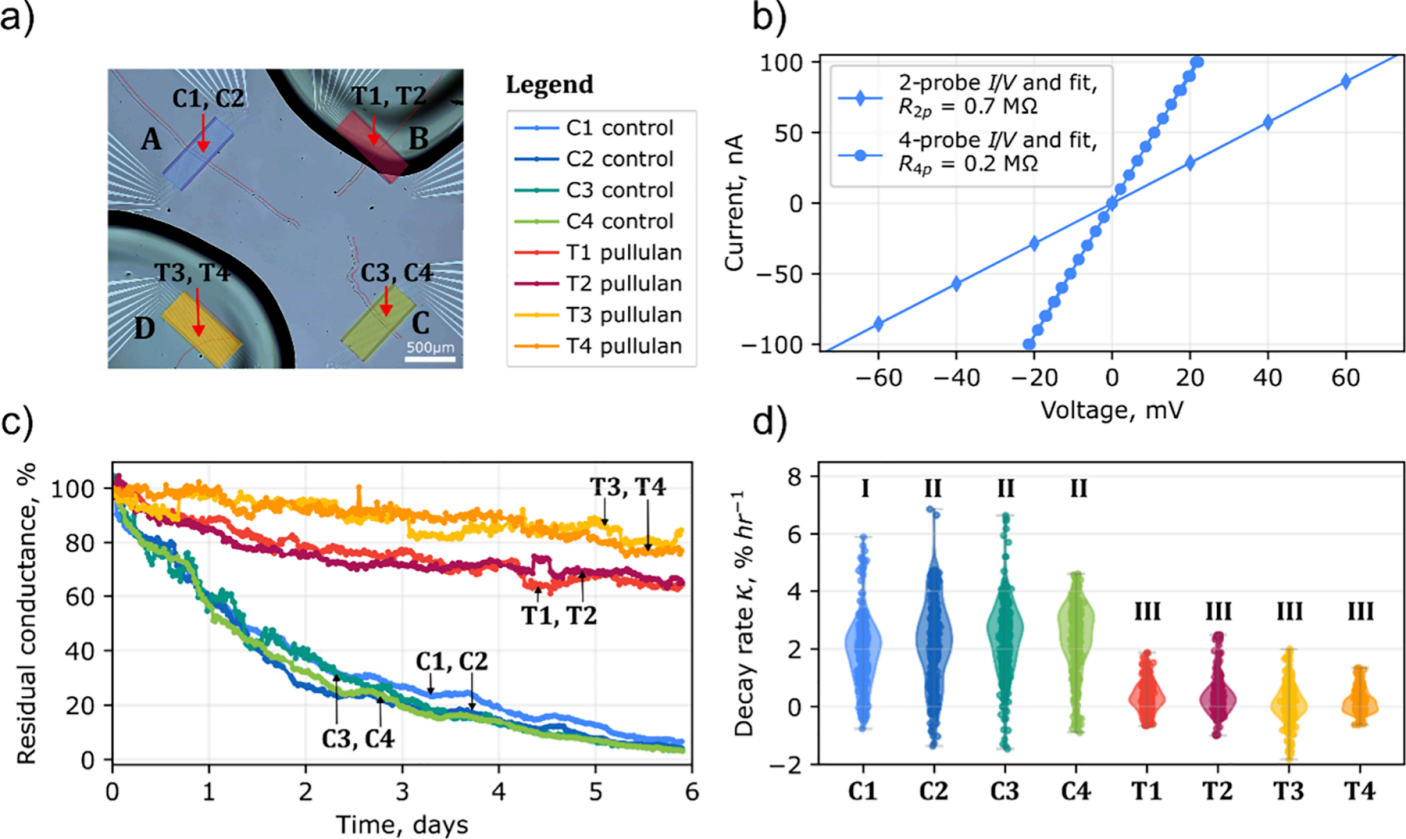
(a) Micrograph of fiber skeletons of cable bacteria (the red lines and arrows trace the filaments) placed on gold electrodes and coated with a pullulan film on pads B and D. (b) Representative examples of two- and four-probe *I*/*V* curves (recorded at the beginning of the stability experiment from sample C1). (c) Dynamics of residual conductance of fiber skeletons derived from the *I*/*V* curves. (d) Violin plots of the conductance loss rates derived from smoothed resistance values that correspond to data from panel (c). Violin plots with different indices are significantly different (*p* < 0.05).

### 3.1 Impact of Pullulan Coating on Conductance in Ambient Air

Upon exposure to ambient air, a loss of conductance was observed in both the pullulan-coated and control samples, but the decrease was much higher and more rapid in the controls (Figure 3c). At the end of the sixth day, the pullulan coating preserved more than 60% of the initial conductance, while the controls degraded to less than 10% of their initial values.

Interestingly, the residual conductance did not decrease monotonously but showed variation at different time scales. At specific time points, the conductance decreased or increased with a marked jump (e.g., segment T2 at minute 6284, T3 at minute 997, C3 at minute 600; Figure S1 in the Supporting Information). We reckon these jumps could be due to settling of the filaments, inducing a sudden change in the contact resistance. In addition, we noted periods of slower and faster degradation (the time-dependent representation of the conductance loss rates is given in Figure S3), with a typical period of ∼24 h. We attribute this to daily variations in temperature and relative humidity (RH), which could, in turn, cause structural changes in the samples such as shrinking and swelling, influencing the contact to the electrodes. This latter effect was more pronounced in the control samples, while marked jumps in conductance occurred both in pullulan-coated and in controls. The ability to partially recover conductance has also been observed in other studies on cable bacterial filaments under different experimental conditions. For example, when freshwater cable bacteria were placed on interdigitated electrodes and transferred from ambient air to N_2_, the sample filament regained around 20% of its initial conductivity value.^60^

The marked difference between treatments is reflected in the conductance loss rates, κ (violin plots in Figure 3d). The uncoated samples showed significantly higher median κ values and possessed a wider distribution than samples coated with pullulan. The control samples showed a median conductance loss rate of κ = 2.31% h^–1^ with interquartile range [1.48–3.04] % h^–1^ (*N* = 1490), while that of pullulan-coated skeletons was 10-fold smaller κ = 0.19 [−0.1–0.52] % h^–1^ (*N* = 1490). The conductance loss rates of all of the individual segments that participated in the experiment are summarized in Table 1 and Table S1.

**Table 1:** Resistances, Conductivity, and Conductance Loss Rates κ of Pullulan-Coated and Control Fiber Skeletons^a^

segment	*R*_2p_(*t* = 0), kΩ	*R*_*i*_(*t* = 0), kΩ	*R*_c_(*t* = 0), kΩ	σ_F_(*t* = 0), S cm^–1^	κ, % h^–1^
T1	909.9 ± 2.4	325.5 ± 0.4	584.4 ± 2.5	4.172 ± 0.005	0.28 [−0.01–0.65]
T2	1133 ± 3	337.7 ± 0.7	796.2 ± 2.8	4.021 ± 0.009	0.26 [−0.02–0.56]
T3	4422 ± 22	477.9 ± 6.2	3944 ± 22	3.553 ± 0.046	0.09 [−0.18–0.46]
T4	2539 ± 8	1001 ± 3	1538 ± 8	1.696 ± 0.006	0.12 [−0.12–0.36]
C1	692.5 ± 1.2	215.4 ± 0.9	477.1 ± 1.5	5.517 ± 0.024	1.98 [1.18–2.48]
C2	625.3 ± 1.8	151.1 ± 0.7	473.7 ± 1.9	7.838 ± 0.035	2.27 [1.36–3.13]
C3	8445 ± 55	3716 ± 21	4730 ± 58	0.411 ± 0.002	2.56 [1.67–3.02]
C4	2633 ± 16	1545.4 ± 3.2	1087 ± 16	0.989 ± 0.002	2.53 [1.88–3.2]

a Codes: *R*_2p_(*t* = 0), *R*_*i*_(*t* = 0), *R*_c_(*t* = 0) are the initial two-probe, four-probe (intrinsic), and contact resistances, respectively; σ_F_(*t* = 0) is the initial intrinsic fiber conductivity; κ is the resulting median conductance loss rate with its first and third quartiles

The conductance loss rate was not normally distributed (Shapiro–Wilk test), which necessitated nonparametric statistical tests for pairwise comparison. The pullulan-coated treatments were all significantly different from the control samples (Dunn’s posthoc test, *p* < 0.05) and not significantly different from each other. Control segment C1 was the most stable in the control group and differed not only from the pullulan-coated but also from the other control segments. Such variations in stability presumably originate from differences in the chemical extraction, which can act nonuniformly across the filament’s length.^4^ The length of the fiber skeleton segment that was electrically investigated had no significant influence on the conductance loss rate. The control segments C1 and C2 of filament A (the length of each segment is 6 μm) degraded similarly to C3 and C4 of filament C (length 10 μm). The same was true for pullulan-coated segments T1, T2, T3, and T4 of lengths 8 and 12 μm. Furthermore, similar conductance loss rates were observed in fiber skeleton segments spaced by 100 μm (Figure S4 and Table S1 in the Supporting Information).

The observed consistency of the conductance loss rates across filaments of varying lengths and initial conductances illustrates the practical applicability of this metric for designing bioelectronic systems based on cable bacterium fibers. However, the conductance loss rate value κ may depend on the experimental procedure and the specific type of cable bacteria examined. A previous study has reported faster conductance decay, with κ reaching up to 20% h^–1^, though the same fiber skeleton extraction protocol was applied.^6^ This variability can be induced by several factors. Foremost, the fiber skeletons still embed way more material than the actual conductive fibers, which can cause variations in the resistance. Future work must decouple the conductance changes of the core conductive fiber component from the influence of the surrounding structures remaining after filament extraction. Likewise, any variation in the extraction protocol itself should be critically evaluated (e.g., bacterial strain, the chemicals used for extraction, and the environmental conditions). Finally, deeper insight into the molecular composition of the conductive component and the underlying fundamental conduction mechanism will also be crucial to constrain the factors that influence the conductance loss rate.

To verify the possibility of long-term storage, the stability experiment was later repeated with an exposure delay. For this, a second MEA with fiber skeletons was prepared at the same time as the first sample batch but stored for 5 months in an argon atmosphere in a glovebox before exposure to ambient air. Notably, even after this long storage period, the fiber skeletons still exhibited high conductivity comparable to the initial one (Figure S5 and Table S1), indicating that Ar does not markedly influence the conductance of cable bacteria. After exposure to air, the second experiment showed similar results to the first: the decay of the conductance was considerably slowed down by pullulan coverage.

### 3.2 Distinguishing the Contributions from Contact and Intrinsic Resistances

The results above are based on two-probe measurements, meaning the resistance reflects the contribution of both the intrinsic sample resistance as well as the contact resistance between electrodes and filaments, i.e., *R*_2p_ *= R*_*i*_ + *R*_c_. In previous work, it has been noted that the contact resistance *R*_c_ can account for 12% to 79% of the total resistance, depending on the size of the electrodes and quality of the contact.^9^ Therefore, the ability to extract *R*_*i*_ and *R*_c_ separately enables a more detailed evaluation of the protective properties of pullulan. To this end, we recorded *I*/*V* curves in a four-probe configuration, with external electrodes supplying the current. The resistance across the internal electrodes designates the intrinsic sample resistance, *R*_4p_ = *R*_*i*_,^58,59^ while the difference between two-probe and four-probe provides the contact resistance, *R*_c_ *= R*_2p_ *– R*_4p_. The fiber conductivities σ_F_ calculated from *R*_*i*_ (0.4–7.8 S cm^–1^) align with the previously reported values^5,6,9^ and demonstrate the functionality of cable bacterium filaments as excellent biological conductors (Table 1 and Table S1). It is notable that the conductance loss rates are independent of the initial σ_F_ (Table 1).

Figure 4 illustrates how the contact and intrinsic filament resistances evolved over time for samples with and without a pullulan coating. Initially, the contact resistance made up 41–76% of the total resistance in the control samples and 61–89% in the pullulan-coated samples. Accordingly, the contact resistance can take up a considerable proportion of the total resistance, in line with previous assessments.^9^ After 6 days of exposure to air, both *R*_i_ and *R*_c_ increased in the control samples, although the former effect was more pronounced (*R*_*i*_ increased by a factor 60 ± 22 (*N* = 4), while *R*_c_ increased by 16 ± 5 (*N* = 4)). As already discussed above, the total resistance of the pullulan-coated segments increased much less compared to the controls, with comparable responses for contact and intrinsic resistance (*R*_c_ increased by a factor 1.5 ± 0.2 (*N* = 4); *R*_i_ increased by 1.3 ± 0.3 (*N* = 4)). This indicates that the pullulan coating not only prevents the degradation of the contact but also effectively preserves the intrinsic fiber conductivity.

**Figure 4:**
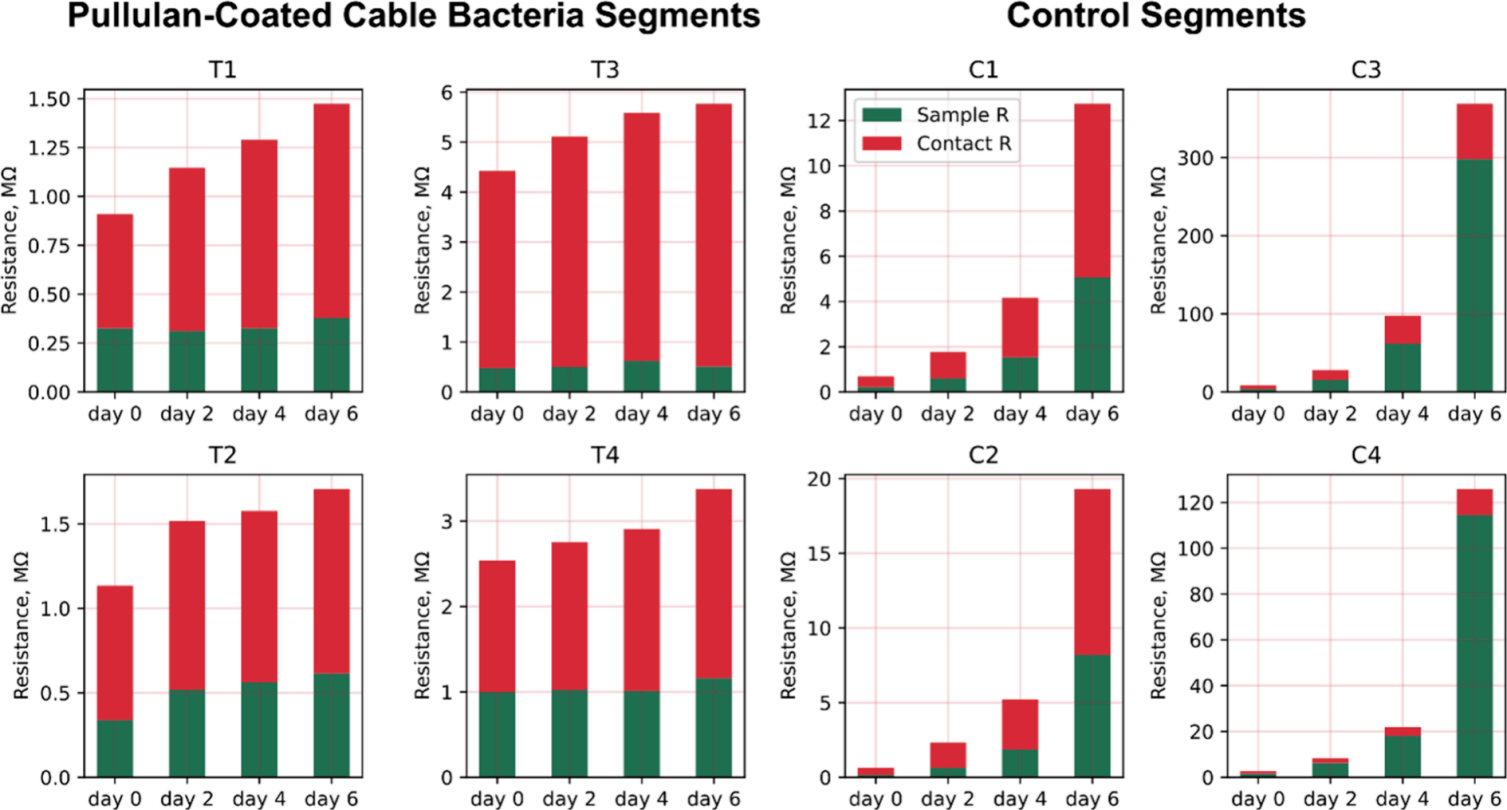
Changes in resistance of the measured fiber skeleton segments decomposed into the intrinsic resistance of the sample *R*_*i*_ (green) and the resistance of the contact *R*_c_ (red) for uncoated control samples and pullulan-coated samples. Note the difference in scale between the graphs.

Our results show that the decay of cable bacterial conductance can be slowed by creating an impermeable barrier to oxygen. At the same time, they provide some insight into the mechanism that causes conductance decay in the uncoated samples. If we assume that the reaction of oxygen with a cofactor molecule follows first-order kinetics and that conductance scales with the concentration of unreacted cofactors (see the Supporting Information for model derivation and discussion), then we expect the conductance *G* to show an exponential decrease with time (i.e., d*G*/d*t* = *–k·G*). For the uncoated controls, an exponential relation *G*(*t*) = *G*_0_ *·*exp­(−*kt*) provides a good fit through the data (low RMSE) and results in a reactivity constant *k* = (7.35 ± 0.88)·10^–6^ s^–1^ (*N* = 4), which can also be represented as a conductance loss rate κ = 2.65 ± 0.32% h^–1^ (Figure 5). Accordingly, the proposed model explains the observed exponential decrease well, but it should be noted that other reaction mechanisms might also provide an exponential conductance decay.

**Figure 5:**
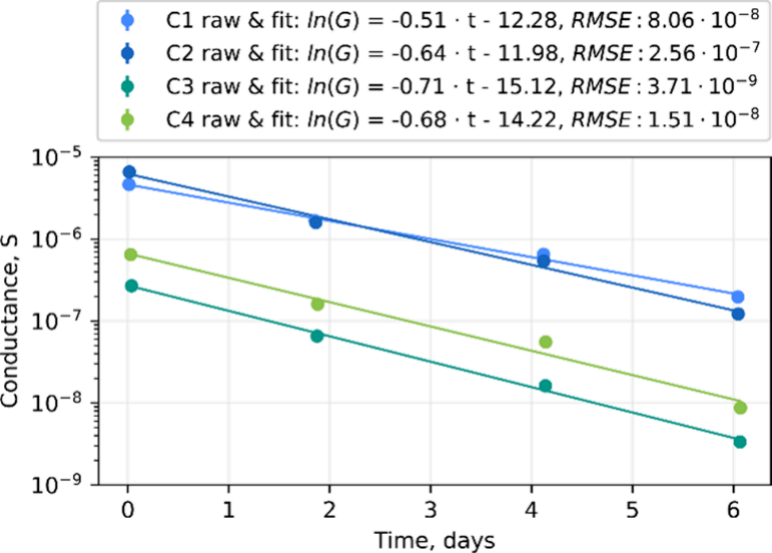
Intrinsic conductance decay of the uncoated control samples in ambient air and the exponential fit that corresponds to the assumed first-order reaction between the cofactor and oxygen. The root-mean-square error (RMSE) indicates the quality of the fit in S units.

At the end of the experiment (after 6 days), the conductance loss in the pullulan-coated group is not fully terminated (Figure 3c), as most likely a small flux of oxygen is sustained by diffusion through the film. The pullulan films used here varied in thickness from 13 to 67 μm. The barrier function can be further enhanced by implementing an enlarged film thickness (e.g., by increasing the viscosity of pullulan solution). Likewise, safeguarding the adhesion of the pullulan film to the sample surface will also be essential. During some of our preliminary tests, the film detached from the MEA, likely due to the residual mechanical stress that accumulates in the film during the drying process. Treating the surface with a silane containing an epoxy group, such as 3-glycidoxypropyl trimethoxysilane (GOPTS), could facilitate cross-linking with the hydroxy group of the pullulan, ensuring reliable film adhesion.^61^

Additionally, the pullulan films are permeable to water vapor, which is readily absorbed.^35,43^ The moisture content, in turn, alters the physicochemical properties of the protective film and affects its oxygen-barrier performance.^62^ For instance, one study evaluated the barrier characteristics of a thin 10 wt % pullulan film (thickness 1.3 μm) and found that increasing the relative humidity from 0% to 80% caused the oxygen transmission rate to rise 10-fold (from below 10 to above 110 mL·m^–2^·day^–1^).^63^ Moreover, water absorption on the outer surface of the filament can form an additional barrier for electric currents, impairing contact at the electrode interface. This may explain why, in pullulan-coated samples, the relative increase in contact resistance slightly exceeded the intrinsic resistance (Figure 4). Therefore, to ensure the stable operation of the cable bacteria-based devices in future applications, pullulan should be best combined with other eco-friendly polymers in multilayered films or hybrid coatings that enhance the resistance to the water vapor such as pectin,^46^ rice wax,^43^ pea protein isolate,^47^ or whey protein isolate with nano-SiO_2_.^44^

### 3.3 Role of Humidity in Cable Bacteria Conductivity Degradation

Apart from O_2_, the material properties of both inorganic and organic conductors can also be significantly affected by relative humidity. At elevated RH levels, many metal contacts are prone to oxidation and corrosion,^64,65^ while protective coatings that have come into contact with water may delaminate.^24^ Likewise, the intrinsic conductivity of organic polymers can be impaired by water absorption, as is the case of PEDOT:PSS. The hygroscopic nature and acidity of the PSS component contribute to the gradual degradation of device performance over time.^66,67^ Initial considerations into the impact of RH on conductance in cable bacteria revealed that short-term exposure of 30 min has no apparent influence on fiber conductivity.^54^ However, the long-term consequences of environmental humidity on these bioconductors have not been investigated until now.

In our stability experiments, the uncoated fiber skeletons showed characteristic periods of fast and slow degradation (Figure 3c). We noticed that these periods corresponded to daily changes in temperature and RH (Figure S3 in the Supporting Information), where an increased RH was correlated with conductance loss. To test the hypothesis that RH impacts the conductance loss rate, an additional experiment was performed after the first experimental phase had finished. The MEA chip with samples was transferred to a desiccator containing silica gel, a drying agent that absorbs water molecules from the air. As a result, the humidity inside the desiccator gradually decreased (10% after 7 h, 6% after 1 day, and 1.5% after 5 days). Sample C1 exhibited the highest conductance by the end of the first experimental phase and was therefore selected among the other samples in the control group (Figure 5). The two-probe resistance of the sample was measured as the MEA was transferred in and out of the desiccator three times (Figure 6).

**Figure 6:**
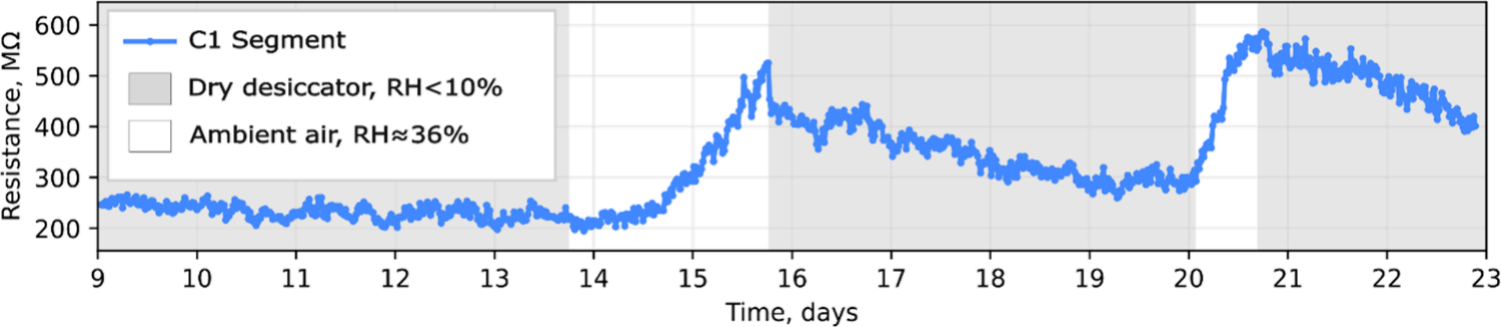
Two-probe resistance dynamics of the uncoated fiber skeleton segment C1 during transfer between the dry desiccator with silica gel and the ambient environment. The time tracking was continued from the start of the experimental session, when the samples were first exposed to ambient air.

The observed dynamics of fiber skeleton resistance reveal that, upon exposure to ambient humidity levels (RH ∼ 36%), the resistance rapidly increased. Inside the desiccator (RH < 10%), however, conductance degradation stopped and even partially reversed. One reason for the observed conductance recovery can be the removal of water molecules from the interface between the filaments and the electrodes in a dry environment, which could cause a decrease in contact resistance. Additional measurements on another sample in a four-probe configuration showed a similar stabilizing effect of RH decrease, although the recovery was less prominent (Figure S6 in the Supporting Information).

It is well established that the degradation of cable bacterium filaments stops in an oxygen-free atmosphere,^6^ and therefore, filament processing and electrical characterization are preferably carried out under an inert atmosphere.^5,9,60,68^ The above results, however, indicate that the conductance decay is the consequence of the conjoint effect of both oxygen and humidity. The removal of one of these two factors allows the preservation of the conductive properties. Thus, the sample conductance remained stable in the anaerobic chamber (Figure S8a), where oxygen was removed with a palladium chloride catalyst. This catalyst causes hydrogen and oxygen molecules to form water molecules.^69^ As a result, the anaerobic chamber maintains high RH levels (55–60%), which does not seem to hamper conductance. Likewise in the desiccator (Figure 6 and Figure S8b), one solely removes water vapor from the air by the silica gel, while the oxygen concentration remains unaffected.^70^

## 4 Conclusions

Due to the high conductivity, the periplasmic fibers in cable bacteria show promise as a biobased material for flexible and transient electronics. Yet, this requires long-term stability of the conductance under application conditions. Our experiments confirm that the conductance of the fiber network in cable bacteria is strongly affected by ambient oxygen, resulting in degradation over hours to days (Figure 3). A shielding from O_2_ however helps to preserve the conductive properties. Foremost, storage under an oxygen deficient atmosphere (<20 ppm of O_2_) enables to retain a stable conductance over months. Likewise, we find that a pullulan coating can significantly slow down the oxidative aging of the conductive fiber network in cable bacteria, lowering the conductance loss rate by a factor of 10. Four-probe measurements revealed that the coated samples not only ensure more stable contact with the electrodes but also effectively conserve the intrinsic conductivity. This demonstrates that pullulan films comprise a suitable coating for organic material-based electronics due to their ability to limit oxygen transport.

We also observed an important role of relative humidity on the conductance of the fiber network in cable bacteria. Previous studies indicated that humidity does not have an instantaneous effect on the conductivity. Yet here, we show that over extended periods of time, the conductance deterioration only occurs when both humidity and O_2_ are conjointly present in the atmosphere. The conductance decay was prohibited in the desiccator containing a moisture-absorbing agent at the ambient O_2_ level (Figure 6). The same was true in the reversed conditions in the anaerobic chamber with [O_2_] < 20 ppm and RH = 55–60% (Figure S8a).

Our observations suggest that the degradation of conductivity in the fiber network of cable bacteria requires the simultaneous presence of two factors: oxygen and moisture. Eliminating either factor significantly enhances the stability of the conductance. This insight provides clear guidance for technological protection. Pullulan significantly retards the O_2_ transport, thus considerably conserving the cable bacteria conductance. However, its hydrophilic nature causes the film to become plasticized at elevated humidity, thereby increasing the oxygen transmission rate by an order of magnitude.^63^ Therefore, hybrid coatings combining pullulan with water-resistant materials could be investigated in the future to provide long-lasting, effective protection for biobased electronics.

## Supplementary Material



## Notes

Figure S1, fiber skeletons residual conductance dynamics on short time scale; Figure S2, profilometry of the pullulan film; Figure S3, dynamics of the conductance loss rates; Figure S4, conductance stability of long pullulan-coated fiber skeleton segments; Figure S5, replication of conductance stability experiment; Figure S6, replication of measurements demonstrating the relative humidity influence on samples stability; Figure S7, control *I*/*V* profiles of pullulan film on blank electrodes; Figure S8, *I*/*V* profiles showing stability of cable bacteria samples in either dry or oxygen-free environments; Figure S9, photo of the experimental setup; Figure S10, layout of the microelectrode arrays; Table S1, summary of the conductance stability for all samples that participated in the experiment (PDF)
