# Supplementary material for: Pullulan Coating Preserves High Conductivity in Cable Bacteria Wires
Source: ACS Appl Bio Mater. 2026 Feb 11;9(5):2591–601. doi: 10.1021/acsabm.5c02310 (PMC12958336; doi:10.1021/acsabm.5c02310)
Supplement: Supplementary file 1 [file mt5c02310_si_001.pdf]

Supporting Information

Pullulan Coating Preserves High Conductivity in Cable Bacteria Wires

Anastasia Gerzhik,[1,2] Dmitrii Pankratov,[3] Silvia Hidalgo Martinez,[3] Filip J.R. Meysman,[3] Andreas Offenhäusser,[1,2] Dirk Mayer*,[1]

[1] Institute of Biological Information Processing (IBI-3), Forschungszentrum Jülich, Jülich 52428, Germany
[2] Faculty I, RWTH Aachen University, Aachen 52062, Germany
[3] Geobiology Research Group, Department of Biology, University of Antwerp, Universiteitsplein 1, Wilrijk 2610, Belgium

*Author to whom any correspondence should be addressed.

E-mail: dirk.mayer@fz-juelich.de

1 Chemical Fixation of Cable Bacteria for Focused Ion Beam Scanning Electron Microscopy (FIB-SEM)

The FIB cross-sections were obtained by chemically fixing the native cable bacteria based on a previously reported protocol by Cornelissen *et al.* [S1]. Briefly, after an initial wash step, native filaments from a wild, non-monoclonal culture of Candidatus Electrothrix gigas were placed on a poly-L-lysine (PLL)-coated substrate. The latter is a polycation that enables the sample to adhere to the surface via electrostatic interactions via the negatively charged membrane of the cable bacteria similar to the neuronal cultures as in Stil *et al.* [S2]. The Au-coated Si/SiO_2 wafer was used as the base substrate material. It was cleaned using a hand torch, then incubated for 60 min at room temperature (RT) in a solution containing 10 µg/mL PLL and 4 µg/mL gelatin in Hank's balanced salt solution (all from Merck KGaA, Darmstadt, Germany). Following the PLL incubation, the substrate was rinsed twice with Hank's solution and twice with Milli-Q water.

The substrate with the deposited cable bacteria was incubated in freshly prepared fixative buffer: 2% paraformaldehyde, 2.5% glutaraldehyde in 0.15 M sodium cacodylate) with a pH 7.4 at RT for 30 min. Then the sample was washed 5 times in 0.15 M cacodylate buffer for 3 min each followed by incubation in 1% osmium tetroxide (OsO_4) (EMS, Hatfield, PA, USA), 0.5% potassium ferrocyanide (Merck KGaA, Darmstadt, Germany) in 0.15 M cacodylate buffer for 40 min at RT. At the next step, the sample was incubated in OsO_4 (1% OsO4 in Milli-Q water) for 40 min at RT. Then the sample was washed in Milli-Q water five times for 3 min and incubated overnight at 4°C in 1% uranyl acetate (EMS, Hatfield, PA, USA). The uranyl acetate was removed by washing in Milli-Q water five times for 3 min and then incubated in 0.15% solution of tannic acid in Milli-Q water (Merck KGaA, Darmstadt, Germany) on ice for 3 min. Then the sample was dehydrated using solutions of increasing ethanol concentration (30, 50, 70, 90%, 2 × 100%), for 3 min each at 0 ºC. Infiltration with resin was done through a series of incubations in ethanol:resin mixtures (EMS, Hatfield, PA, USA) at the following ratios: 3:1, 2:1, 1:1, 1:2 and 1:3. All incubations were for two hours except the 1:1 ratio, which was done overnight. This was followed by three incubations in pure resin: the first overnight, and the next two for two hours each. The final washing was done using pure ethanol.

Prior to FIB, the resin block containing cable bacterium filaments was mounted on aluminum SEM stubs and coated with ∼5 nm of platinum. FIB cross-sectioning was performed using the FEI Helios Nanolab 600 (Hillsboro, Oregon, United States). A focused gallium ion beam was employed to selectively remove material from the sample, thereby enabling direct access to the cross-section for imaging. The SEM images were recorded using the backscattered electron detector at 3 kV accelerating voltage, 86 or 690 pA beam current, 4.1 mm working distance and 52° tilt.

2 A Simplified Model for Cofactor Oxidation in Cable Bacteria

We assume that the conduction loss in the periplasmic fiber structures of cable bacteria is linked to the oxidation of a cofactor responsible for conduction (represented as *Co*), according to the following reaction equation:

$$Co + O_2 \rightarrow Cox \tag{S1}$$

The reaction rate *v* depends on concentration of both reactants (oxygen and unreacted cofactor) and can be written as the rate expression:

$$v = -\frac{d[Co]}{dt} = -\frac{d[O_2]}{dt} = \frac{d[Cox]}{dt} = r[Co][O_2] \tag{S2}$$

In this, *r* represents the kinetic rate constant. We assume the conductance linearly scales with the concentration of unaffected cofactor, i.e., $G = a[Co]$

$$\frac{dG}{dt} = a\frac{d[Co]}{dt} = -a \cdot r[Co][O_2] = -r \cdot G[O_2] \tag{S3}$$

Molecular diffusion is an efficient transport mechanism over short distance (Crank [S3]), so we expect O_2 to very quickly diffuse within the fiber skeleton material, which is ~300 nm thick (Cornelissen et al. [S1]). Assuming an aqueous environment in the fibers, diffusion operates on a time scale of $\tau = L^2/2D \sim 20$ microseconds over such a small distance $L = 3\cdot10^{-7}$ m with $D = 2\cdot10^{-9}$ m² s^{-1} (Cussler et al. [S4]). Accordingly, O_2 will be quickly replenished, and the oxygen concentration will remain constant and high in the fiber skeleton. As such the expression for the conductance becomes

$$\frac{dG}{dt} = -kG \tag{S4}$$

The solution to this differential equation implies an exponential decrease of the conductance

$$G(t) = G_0 \exp(-kt) \tag{S5}$$

In this, the rate constant $k = [O_2]r$ has the units of inverse time and can be derived by fitting the above relation to the available conductance data. Note that the conductance loss rate κ introduced in the main text provides an estimate for *k* at each time point

$$k = -\frac{1}{G}\frac{dG}{dt} \tag{S6}$$

3 Stability Experiments with Pullulan Coating

3.1 Stability Experiment A (MEA1)

In Experiment A, we investigate the conductance decay for short fiber skeleton segments using MEAs with an interspacing ranging from 6 to 12 µm (the layout is given in Figure S10 and Table S2). The experimental procedure is described in detail in the main text. Four pullulan-coated segments (T1-T4) and four uncoated control samples (C1-C4) were investigated. The experimental results are summarized in the Tables 1 and S1. The plot for the temporal evolution of residual conductance is provided in Figure 3c. Plots focusing on jump events that occur within specific time intervals are depicted in Figure S1. Violin plots summarizing the distribution of the conductance loss rates are given in Figure 3c. The samples T1-T4 are covered with pullulan coating. We determined the thickness of the coating by profilometry in Figure S2.

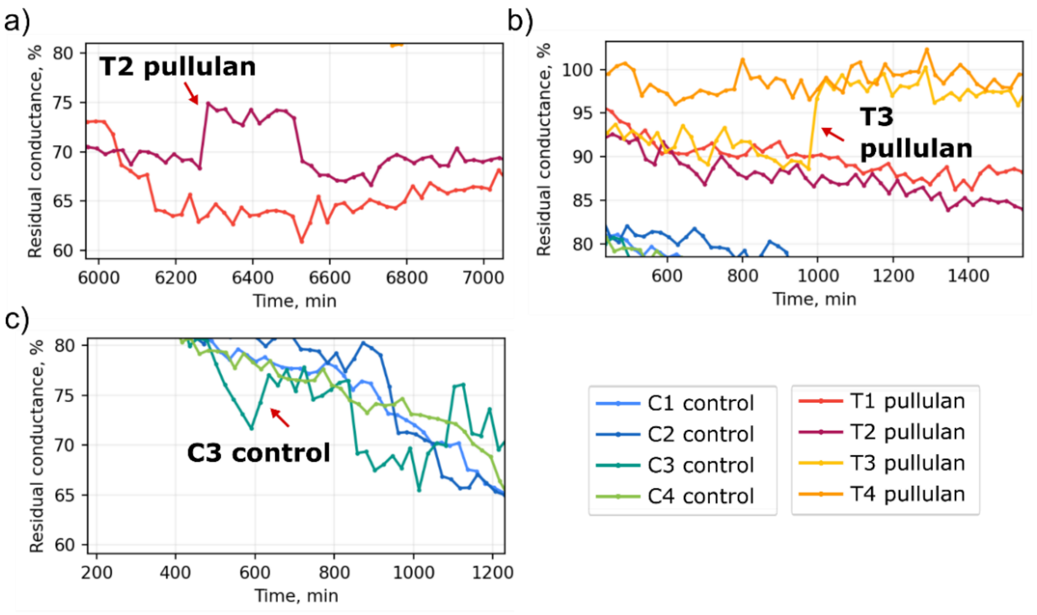

Figure S1. Zoom in on the dynamics of the residual conductance of the fiber skeletons on the MEA1, showing the periods of conductivity recovery (marked by the arrows) for the segments covered with pullulan: (a) T2 and (b) T3, as well as the non-coated control (c) C3.

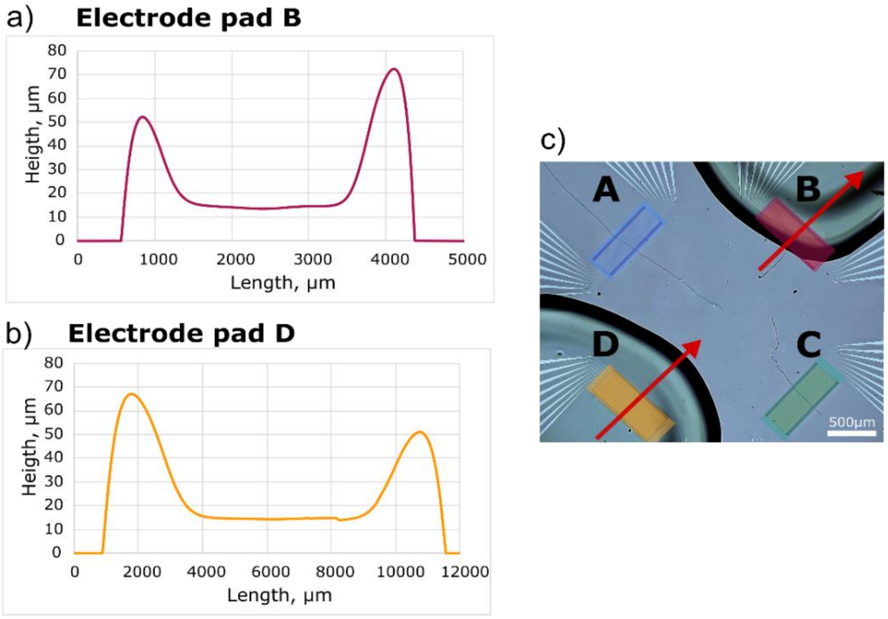

Figure S2. Profilometry of the MEA1 with fiber skeletons coated with the pullulan film. (a) Electrode pad B with segments T1,T2. (b) Electrode pad D with segments T3,T4. (c) Light microscopic image showing the location and direction of the scan.

The conductance loss rates $\kappa = 1/R*dR/dt = -1/G*dG/dt$ were calculated at each time point from the smoothed resistance values $R(t)$ and are given in Figure S3. Periods of slower and faster decay are more pronounced in non-coated samples (Figure S3b) and correspond to daily variations in temperature and humidity (Figure S3a). The impact of temperature on conductance of cable bacteria can be rationalized as an Arrhenius dependence: $G = G_0 \cdot exp(-E_a/RT)$ (Atkins *et al.* [S5], Bonné *et al.* [S6]). However, the observed temperature variations of 3 °C could yield only in conductance change rate κ = 0.1-0.2 % hr^{-1} given the activation energy for electron transport in cable bacteria E_a = 40 meV (Bonné *et al.* [S6]). Thus, thermal activation of electron

transport does not account for the observed variations of 4 % hr^{-1}. The reaction constant for the conducting cofactor oxidation is also directly influenced by temperature following an Arrhenius equation with yet unknown E_a. However, an activation energy as high as 2 eV (the typical E_a for the oxidation of organic substances (Tsang and Hampson [S7])) would only change the rate by a factor of two, which is insufficient to explain the broad range of registered decay rates.

Therefore, we hypothesize that relative humidity influences the decay rate with a 12-hour delay. The corresponding Pearson correlation coefficients between RH and conductance loss rates of control samples are 0.55 (strong correlation for C1), 0.42 (moderate C2), 0.45 (moderate C3) and 0.32 (weak C4) respectively. For pullulan-coated samples, the absolute value of the Pearson correlation coefficient is below 0.3 and therefore can be considered as negligible. The hypothesis that humidity plays an important role in the degradation of cable bacteria was later tested in an O_2-saturated, dry environment (Figures 6 and S6).

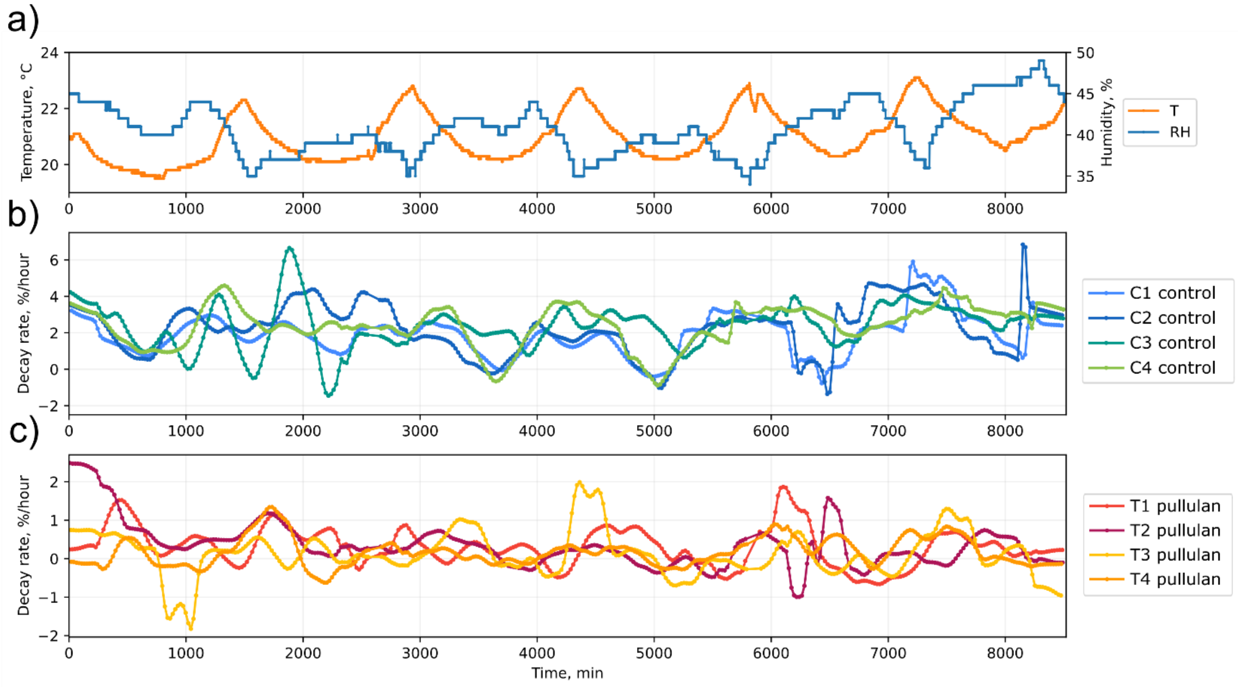

Figure S3. Dynamics of the sample conductance loss rate κ and corresponding temperature and relative humidity recordings. Periodic changes in the decay rate of the control samples are positively correlated with humidity when the 12-hour delay in the decay rate response is considered. Note the difference in scale on planes b) and c)

3.2 Stability Experiment B

To confirm the results obtained in Experiment A and to investigate the conductance decay for longer fiber skeleton segments, we repeated the stability experiment using 100 µm wide electrodes with an interspacing of 100 µm. The substrate preparation procedure, filament extraction and experimental conditions were identical as in Experiment A. Chips with electrodes were prepared in the cleanroom by evaporating 20 nm Ti (adhesion layer) and 120 nm Au on the Si/SiO_2 substrates. The fiber skeleton extraction was conducted in the anaerobic chamber, and 20 µl of 10 wt% pullulan in MilliQ solution was applied. After drying for 24 hours, samples were brought to the ambient air and characterized by continuous *I/V* profile recording using a PalmSens4 potentiostat with MUX8 multiplexer in 2-probe configuration from one of the pairs of neighbouring electrodes.

Two pullulan-coated segments (T5,T6) and two control samples (C5,C6) were investigated (Figure S4a). The evolution of the residual conductance is displayed in Figure S4b. Violin plots summarizing the distribution of the conductance loss rates are given in Figure S4c. The experimental results are summarized in Table S1. The estimated values of the conductance loss rates are highly similar to those of shorter fiber skeleton segments (6-10 µm) investigated in Experiment A.

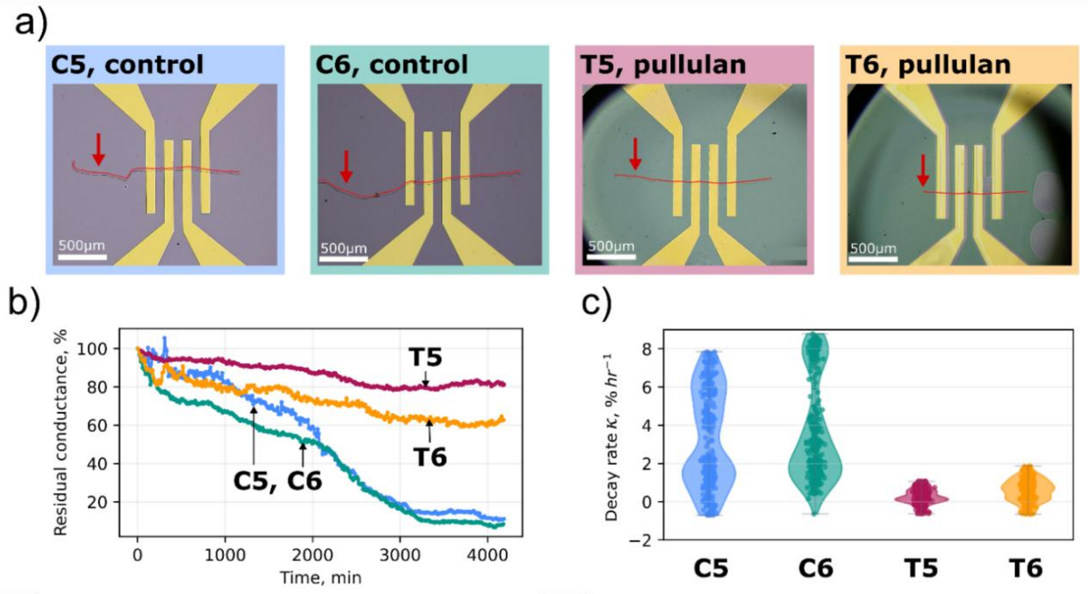

Figure S4. Impact of pullulan coating on conductance stability for long segments. (a) Fiber skeletons (red lines and arrows trace the filaments) are placed on substrates with gold electrodes with 100 µm interspacing. Pullulan-coated (T5, T6) and control samples (C5,C6) are shown. (b) Temporal evolution of the residual conductance derived from 2-probe *I/V* profiles recorded in ambient atmosphere (T=20-23 °C, RH=35-50%, $[O_2]$=21%). (c) Violin plots of the conductance loss rates κ.

3.3 Stability Experiment C (MEA2)

The samples for Experiment C were prepared alongside those of Experiment A (Figure S5a). Fiber skeletons were extracted, placed on the electrodes (MEA2) and coated with pullulan at the same day as in Experiment A. The layout and fabrication of MEA2 is identical to that of MEA1 (see materials and methods in the main paper, Figure S10 and Table S2). After the pullulan coating, samples on MEA2 were stored for 5 months in a glove box (inert Ar atmosphere) prior to measurements in ambient atmosphere.

Three pullulan-coated segments (T7-T9) and three control samples (C7-C9) were investigated. The evolution of the residual conductance is displayed in Figure S5b. Violin plots visualizing the distribution of the conductance loss rates are given in Figure S5c. The experimental results are summarized in Table S1. Notably, "aged" pullulan-coated segments showed the same temporal evolution as freshly prepared samples upon exposure to ambient air (Table S1).

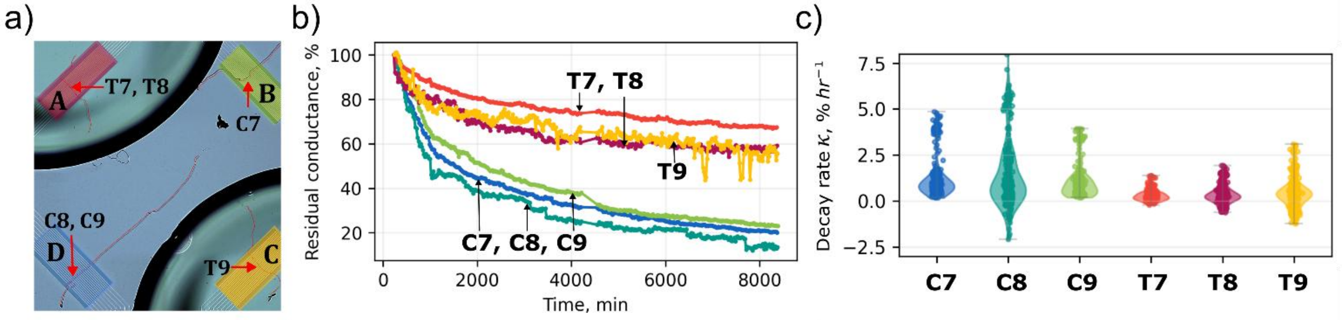

Figure S5. Stability of the pullulan-coated (5µL of 10wt% pullulan in MilliQ) and control fiber skeletons on MEA2. (a) Micrograph of fiber skeletons of cable bacteria (the red lines and arrows trace the filaments) placed on gold electrodes and coated with pullulan film on pads A and C. (b) Dynamics of residual conductance of fiber skeletons derived from the 2-probe *I/V* profiles in an ambient atmosphere (T=19-21 °C, RH=35-50%, $[O_2]$=21%) (c) Violin plots of the conductance loss rates.

3.4 Summary of Conductance Stability Measurements

Table S1. Resistances, conductivity and conductance loss rates of all characterized pullulan-coated and control fiber skeleton segments

Segment	Segment Length, µm	Sample	$R_{2p}(t=0)$, kΩ	$R_i(t=0)$, kΩ	$R_c(t=0)$, kΩ	$\sigma_F(t=0)$, S cm^{-1}	κ, % hr^{-1}
**Pullulan-coated segments**							
**T1**	8	**MEA1**, **Fiber Skeleton 2**, Freshly Extracted. Electrode Set B	909.9 ± 2.4	325.5 ± 0.4	584.4 ± 2.5	**4.172** ± 0.005	**0.28** [-0.01-0.65]
**T2**	8		1133.2 ± 2.7	337.7 ± 0.7	796.2 ± 2.8	**4.021** ± 0.009	**0.26** [-0.02-0.56]
**T3**	12	**MEA1**, **Fiber Skeleton 4**, Freshly Extracted. Electrode Set D	4421.7 ± 21.5	477.9 ± 6.2	3943.8 ± 22.3	**3.553** ± 0.046	**0.09** [-0.18-0.46]
**T4**	12		2538.8 ± 7.8	1000.9 ± 3.3	1538.0 ± 8.4	**1.696** ± 0.006	**0.12** [-0.12-0.36]
**T5**	100	**Substrate 3, Fiber Skeleton 7**, Freshly Extracted.	49632.9 ± 23.1	-	-	**0.171** ± 0.0001*	**0.18** [0.05-0.55]
**T6**	100	**Substrate 4, Fiber Skeleton 8**, Freshly Extracted.	24788.7 ± 19.8	-	-	**0.3424** ± 0.0003*	**0.63** [0.14-1.01]
**T7**	6	**MEA2**, **Fiber Skeleton 9**, 5 months old. Electrode Set A	268.1 ± 0.5	183.0 ± 0.2	85.1 ± 0.5	**6.493** ± 0.007	**0.25** [0.11-0.43]
**T8**	6		3403.3 ± 11.2	3351.5 ± 30.2	51.8 ± 32.2	**0.355** ± 0.003	**0.28** [0.07-0.58]
**T9**	10	**MEA2**, **Fiber Skeleton 11**, 5 months old. Electrode Set C	3837.2 ± 25.8	1407.3 ± 14.3	2429.9 ± 29.5	**1.086** ± 0.011	**0.35** [0.04-0.68]
**Control segments**							
**C1**	6	**MEA1**, **Fiber Skeleton 1**, Freshly Extracted. Electrode Set A	692.5 ± 1.2	215.4 ± 0.9	477.1 ± 1.5	**5.517** ± 0.024	**1.98** [1.18-2.48]
**C2**	6		625.3 ± 1.8	151.1 ± 0.7	473.7 ± 1.9	**7.838** ± 0.035	**2.27** [1.36-3.13]
**C3**	10	**MEA1**, **Fiber Skeleton 3**, Freshly Extracted. Electrode Set C	8445.5 ± 54.5	3715.6 ± 20.9	4729.9 ± 58.4	**0.411** ± 0.002	**2.56** [1.67-3.02]
**C4**	10		2632.8 ± 15.8	1545.4 ± 3.2	1087.4 ± 16.1	**0.989** ± 0.002	**2.53** [1.88-3.2]
**C5**	100	**Substrate 1, Fiber Skeleton 5**, Freshly Extracted.	91511.9 ± 291.0	-	-	**0.0928** ± 0.0003*	**2.30** [1.19-5.72]
**C6**	100	**Substrate 2, Fiber Skeleton 6**, Freshly Extracted.	9450.1 ± 9.5	-	-	**0.8982** ± 0.0009*	**2.67** [1.57-6.09]
**C7**	8	**MEA2**, **Fiber Skeleton 10**, 5 months old. Electrode Set B	1082.5 ± 6.7	376.0 ± 2.0	706.5 ± 7.0	**3.612** ± 0.019	**0.90** [0.60-1.23]
**C8**	12	**MEA2**, **Fiber Skeleton 12**, 5 months old. Electrode Set D	1873.2 ± 9.3	597.2 ± 2.5	1276.0 ± 9.6	**2.843** ± 0.012	**0.86** [0.32-2.29]
**C9**	12		1096.5 ± 3.1	465.7 ± 1.6	630.8 ± 3.5	**3.645** ± 0.012	**0.82** [0.41-1.22]

Codes: $R_{2p}(t=0)$, $R_i(t=0)$, $R_c(t=0)$ – initial 2-probe, 4-probe (intrinsic), and contact resistances; $\sigma_F(t=0)$ – initial intrinsic fiber conductivity; κ – resulting median conductance loss rate with its first and third quartiles.
Conductivities marked with * are calculated from 2-probe resistance (not intrinsic).
The table shading indicates the sample groups that were prepared in the anaerobic chamber alongside each other.

4 Influence of Relative Humidity (RH) on Conductance Decay of the Fiber Skeleton Segment C9

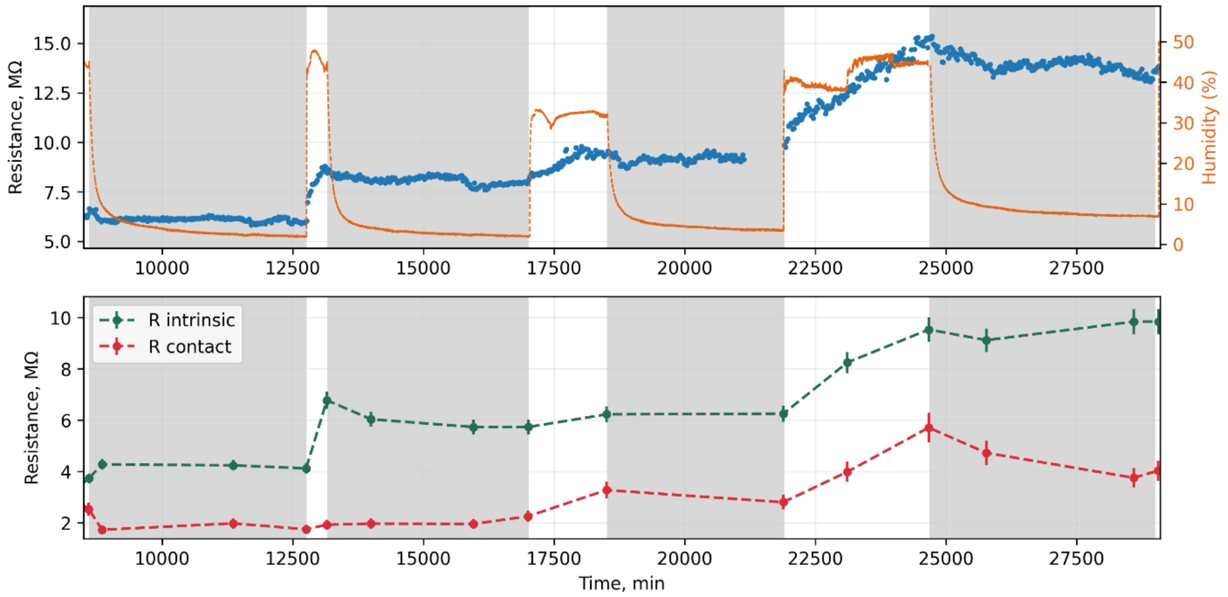

Figure S6. Resistance dynamics of the uncoated fiber skeleton segment C9 (MEA2) during transfer between the dry desiccator with silica gel and ambient environment. The time tracking continued from the start of the experimental session, when the sample was first exposed to ambient air.

5 Control Measurements of Pullulan Film Without Cable Bacteria

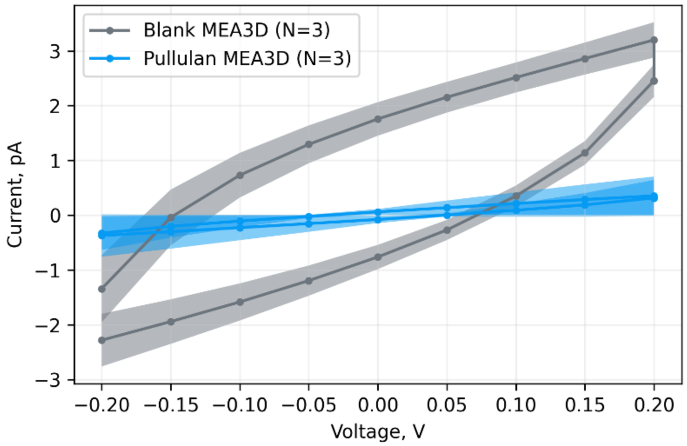

Figure S7. Control *I/V* measurements of the pullulan film formed on blank MEA under the same conditions as coating for the cable bacteria samples. Width of the electrodes: 8 µm, distance: 12 µm. The decreased signal amplitude after the application of the pullulan film indicates its dielectric properties.

6 *I/V* Profiles of Cable Bacteria Fiber Skeletons Preserved in Anaerobic Chamber or Dry Desiccator

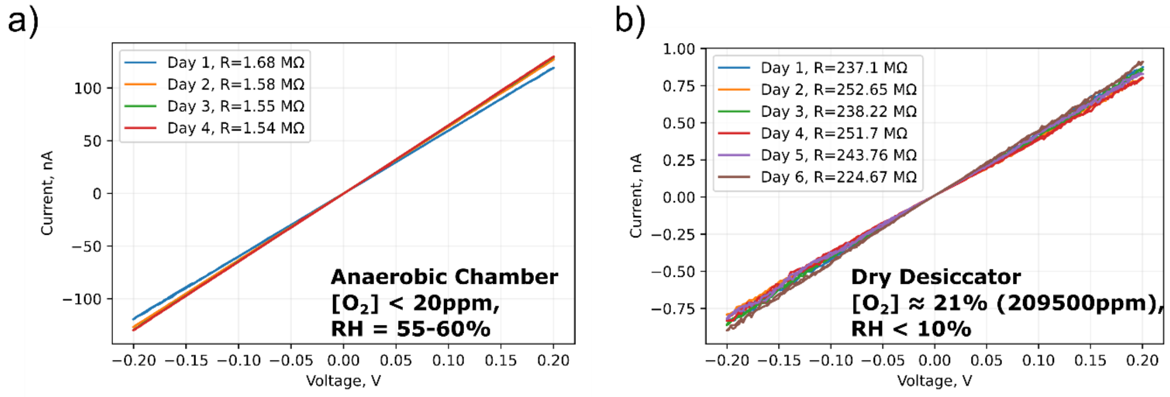

Figure S8. *I/V* profiles of the cable bacterium fiber skeletons demonstrating the stability of conductance within a period of observation of 4-6 days in two controlled environments. (a) An anaerobic chamber in the absence of oxygen, but with the presence of water vapor. Length of the fiber skeleton segment: 100 µm. (b) A desiccator with moisture-absorbing silica gel, resulting in a relative humidity (RH) below 10%, but with an ambient oxygen concentration. Length of the fiber skeleton segment: 6 µm (control segment C1, where day 1 corresponds to the first day in the desiccator (day 8 from the beginning of the experimental session).

7 Experimental Setup for Stability Recordings

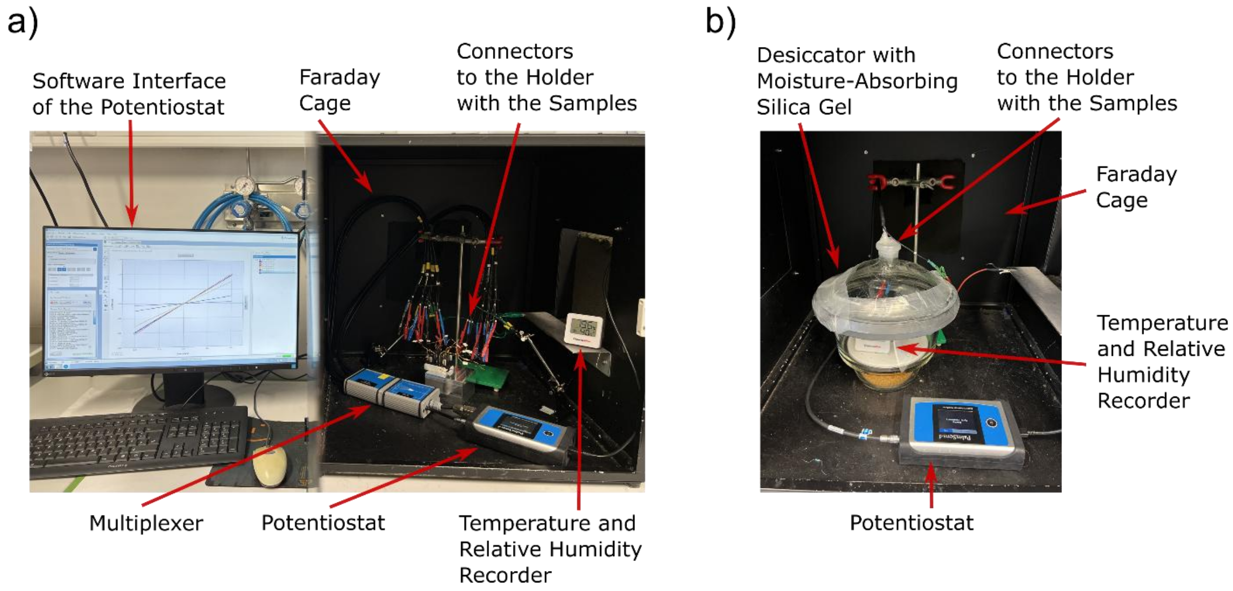

Figure S9. Photo of the experimental setup. (a) Configuration used for the stability measurements of the pullulan-coated and control fiber skeleton segments. (b) Desiccator with silica-gel used for the evaluation of the relative humidity influence on the sample degradation.

8 Layout of the Microelectrode Arrays (MEAs)

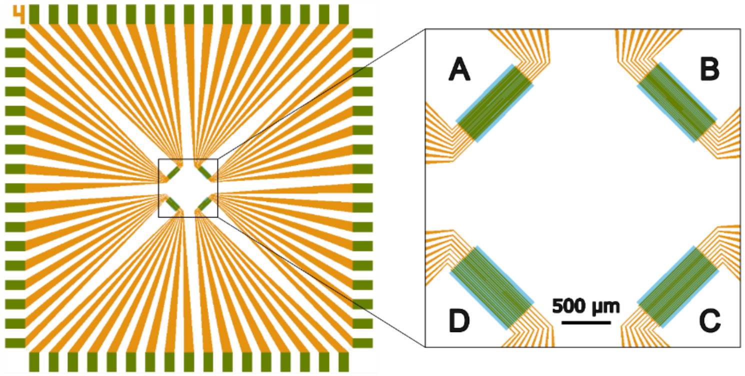

Figure S10. Layout of the microelectrode arrays (MEA1 and MEA2 in experiment). Orange: gold feedlines. Green: location of the openings in the passivation layer (ONONO and Ta_2O_5), which enable contact with the gold for the sample and measurement system.

Table S2 – Dimensions of the MEA

Electrode Pad	Width, µm	Non-conducting interspace, µm	Length, µm	Number of electrodes
**A**	8	6	900	17
**B**	8	8	900	17
**C**	8	10	900	17
**D**	8	12	900	17

References

[S1] Cornelissen R, Bøggild A, Thiruvallur Eachambadi R, Koning R I, Kremer A, Hidalgo-Martinez S, Zetsche E-M, Damgaard L R, Bonné R, Drijkoningen J, Geelhoed J S, Boesen T, Boschker H T S, Valcke R, Nielsen L P, D'Haen J, Manca J V and Meysman F J R 2018 The Cell Envelope Structure of Cable Bacteria Front. Microbiol. 9

[S2] Stil A, Liberelle B, Guadarrama Bello D, Lacomme L, Arpin L, Parent P, Nanci A, Dumont É C, Ould-Bachir T, Vanni M P, De Crescenzo G and Bouchard J-F 2023 A simple method for poly-D-lysine coating to enhance adhesion and maturation of primary cortical neuron cultures in vitro Front. Cell. Neurosci. 17 1212097

[S3] Crank J 1976 The mathematics of diffusion (Oxford: Clarendon Press)

[S4] Cussler E L 2009 Diffusion: mass transfer in fluid systems (Cambridge university press)

[S5] Atkins P W, Ratcliffe R G, De Paula J and Wormald M 2023 Physical chemistry for the life sciences (Oxford University Press)

[S6] Bonné R, Hou J-L, Hustings J, Wouters K, Meert M, Hidalgo-Martinez S, Cornelissen R, Morini F, Thijs S, Vangronsveld J, Valcke R, Cleuren B, Meysman F J R and Manca J V 2020 Intrinsic electrical properties of cable bacteria reveal an Arrhenius temperature dependence Sci Rep 10 19798

[S7] Tsang W and Hampson R F 1986 Chemical Kinetic Data Base for Combustion Chemistry. Part I. Methane and Related Compounds Journal of Physical and Chemical Reference Data 15 1087–279
